# The relationship between government research funding and the cancer burden in South Korea: implications for prioritising health research

**DOI:** 10.1186/s12961-019-0510-6

**Published:** 2019-12-23

**Authors:** Ye Lim Jung, Hyoung Sun Yoo, Eun Sun Kim

**Affiliations:** 1 Technology Commercialization Center, Division of Data Analysis, Korea Institute of Science and Technology Information (KISTI), 66 Hoegiro, Dongdaemun-gu, Seoul, 02456 Republic of Korea; 2 Science and Technology Management Policy, University of Science and Technology, 217 Gajeong-ro, Yuseong-gu, Daejeon, 34113 Republic of Korea

**Keywords:** Disease-specific funding, Burden of disease, Cancer burden, Health research priorities, Government-sponsored programme

## Abstract

**Background:**

In this study, we aimed to assess health research funding allocation in South Korea by analysing the relationship between government funding and disease burden in South Korea, specifically focusing on cancers.

**Methods:**

The relationship between research funding and the cancer burden, measured in disability-adjusted life-years (DALYs), was analysed using a linear regression method over a 10-year interval. Funding information on 25 types of cancer was obtained from the National Science and Technology Information Service portal in South Korea. Measures of cancer burden were obtained from Global Burden of Disease studies. The funding predictions were derived from regression analysis and compared with actual funding allocations. In addition, we evaluated how the funding distribution reflected long-term changes in the burden and the burden specific to South Korea compared with global values.

**Results:**

Korean funding in four periods, 2005–2007, 2008–2010, 2011–2013 and 2015–2017, were associated with the cancer burden in 2003, 2006, 2009 and 2013, respectively. For DALYs, the correlation coefficients were 0.79 and 0.82 in 2003 and 2013, respectively, which were higher than the values from other countries. However, the changes in DALYs (1990–2006) were not associated with the funding changes (from 2005 to 2007 to 2015–2017). In addition, the value differences between Korean and global DALYs were not associated with Korean government research funding.

**Conclusions:**

Although research funding was associated with the cancer burden in South Korea during the last decade, the distribution of research funds did not appropriately reflect the changes in burden nor the differences between the South Korean and global burden levels. The policy-makers involved in health research budgeting should consider not only the absolute burden values for singular years but also the long-term changes in burden and the country-specific burden when they prioritise public research projects.

## Introduction

The funding criteria for national research projects have been an issue of political importance in a variety of countries [1–5]. Over the past decades, there have been inconsistencies in the budget allocation for public research due to the intervention of subjective judgments or political influences from various parties [1, 6]. Since resources are limited, the government’s budget should be properly and effectively distributed to generate the greatest benefits. Especially in the field of health, governmental research should focus more on public goals, such as resolving health challenges that broadly affect the population, because one of the aims of the government is to improve the health condition of the nation as a whole, unlike actors in the private sector (enterprises and corporations), which aim to pursue their own profit [7–12].

In this regard, substantial efforts have been made to establish criteria for health research investment based on rational and objective evidence [13–21]. As a part of this endeavor, Gross et al. conducted an original study in 1999 that analysed disease burden and National Institutes of Health (NIH) funding in order to set up investment criteria grounded on health data [22]. Measures of disease burden such as incidence, mortality, years of life lost (YLLs) and disability-adjusted life years (DALYs) are fundamental indicators that represent public health conditions [23, 24]. The researchers analysed the relationship between these burden measures and the amounts of funding respectively allocated to the various types of diseases. Thereafter, several studies were conducted in other countries, including the United States, United Kingdom, Norway, Australia and China, to examine the disease-specific funding of government-sponsored research projects [25–30].

Despite this progress in global research, however, there has not yet been adequate investigation of the governmental funding in South Korea in relation to public health improvement. The total amount of the Korean government’s research funding in the area of bio/health in 2012 was of approximately 1.66 billion USD (1.87 trillion Korean won (KRW)) and increased to 2.13 billion USD (2.47 trillion Korean won (KRW)) in 2016, with a percentage increase of 32.4% over the past 5 years. When compared to other countries, such as the United States (36.9 billion USD), Germany (1.9 billion USD), Japan (1.4 billion USD) and France (1.2 billion USD), in the same year (2016), the Korean government plays an important role as one of major funders in the world [31]. In addition, when compared to other countries using health gross domestic research and development (R&D) expenditure on health and medical sciences as a percentage of gross domestic product (GDP), South Korea’s health gross domestic R&D expenditure was 0.21% of its GDP in 2016, which is higher than the average among the world’s high-income countries (0.19% of GDP) and also much higher than the average in other regions such as the Western Pacific (0.07%), South-East Asia (0.03%), the Americas (0.03%) and Europe (0.03%) [32]. Notwithstanding such a large-scale investment, there have been few studies that assess whether these research funds were appropriately allocated to meet public health needs.

To address this need, we analysed the correlation between national funding and disease burden in South Korea. Our study focused on cancer since it is the leading cause of death in Korea, accounting for over 79,000 deaths in 2016 [33], as well as the second leading cause of death worldwide, causing 8.7 million deaths in 2015 [34]. In addition, cancer has the advantage of highly accurate values of statistical burden measures since it benefits from a high level of certainty in diagnosis thanks to the use of histological diagnostics (rather than clinical or radiographical diagnostics). Despite the importance of cancer, there has been very little knowledge regarding the allocation of governmental funds according to cancer type, since there have been few studies dedicated to analysing the data on types of cancer [27, 35–40]. In response, we investigated the relationship between governmental research funding and the cancer burden in Korea and examined the changes in their correlation over the last decade. Furthermore, we investigated how the funding distribution reflected long-term changes in the burden and reflected the burden specific to South Korea compared with global values.

## Methods

In order to analyse the relationship between research funding and cancer burdens, two types of data were collected, as follows.

Funding data from 2005 to 2017, categorised by cancer type, were obtained from the National Science & Technology Information Service (NTIS), officially administered by the South Korean government (Ministry of Science and Information and Communications Technology) [41]. The NTIS is a comprehensive database system, the world’s first national R&D information portal that gathers, manages and provides all government-funded research information [42, 43]. It offers details on budgets, contents, duration and outcomes of research projects provided by Korean governmental departments and agencies. To extract data on research funding allocated specifically to various types of cancer, we conducted a keyword search using search fields, including research title, research objective, research keyword and research summary. The research projects containing the main keywords of each cancer type were considered cancer-specific projects, that is, projects specific to that type of cancer. The main keywords used for identifying research funding data by cancer type are shown in Additional file 1: Table S1. In the case of basic research, such as studies on the discovery of biomarkers or the investigation of mechanisms, the research projects were included in the counts for multiple types of cancer because their findings can be utilised in various types of cancers (i.e. amounts of cancer-specific funding were estimated in a non-mutually exclusive manner). Sensitivity analysis was performed while excluding the projects that were counted multiple times from the total funding dataset. To take account of the annual fluctuations in the amounts of funding, we summated the 3-year value from 2005 to 2007, from 2008 to 2010, from 2011 to 2013, and from 2015 to 2017, respectively, and used the summated values of 2005–2007 and 2015–2017 to compare the change in funding over the 10-year interval. The funding amounts in each year from 2005 to 2016 were adjusted for 2017 equivalents in order to remove the effect of general inflation.

The global and South Korean values for the cancer burden in 2003, 2006, 2009 and 2013 (single year values), and the changes in the burden from 1990 to 2006 were acquired from the Global Burden of Diseases (GBD) studies of WHO [34, 44]. GBD systemically measures a variety of disease burden indicators (all-cause mortality, deaths by cause, YLLs, DALYs, prevalence, incidence, life expectancy, etc.) worldwide and its estimates are updated annually. We utilised four measures of burden — incidence, mortality, YLLs and DALYs — for the analyses in this study.

Out of a total of 32 types of cancers according to the GBD classification, there were 25 types of cancers for which South Korean research funding data were available and this data was matched and analysed.

We applied a time lag of 2–4 years between cancer burden and research funding, since lag periods of 2–6 years had been applied for analysis in previous studies [22, 25, 26, 28, 30, 35]. That is, we compared DALYs in 2003 and the sum of the research funding in the period of 2005–2007, DALYs in 2006 and the research funding in 2008–2010, DALYs in 2009 and the research funding in 2011–2013, and DALYs in 2013 and the research funding in 2015–2017. The funding amounts and the burden values were log-transformed for analysis. Comparisons with other studies were also performed under the same conditions by converting the variables to a logarithmic scale in cases where there were variables not presented in log-scale.

‘Google Trends’ was used to analyse the web search intensity of internet users by types of cancer as a public interest variable [45, 46]. The same keywords that were used for searching cancer-specific research funding were also applied to find data on the web search intensities for 25 types of cancer.

Univariate or multivariate linear-regression was performed to evaluate the relationship between the funding level and the disease burden and/or public interest. Correlations were assessed by the Pearson correlation coefficient and Spearman’s Rho at a 95% confidence level.

We used the regression results to calculate the amounts of counterfactual funding for each cancer type, assuming a scenario in which funding is solely determined by disease burden, and then compared these counterfactual amounts to the actual funding amounts. All analyses were performed using the SPSS statistical package (version 20.0, Chicago, Ill, USA).

## Results

The total research budget of South Korea devoted to cancer during 2005–2017 was approximately 1.68 billion USD. In our analysis of 25 types of cancer (which allowed multiple counting of research projects that can be utilised in several types of cancer to multiple cancer types), total funding amounts ranged from 0.3 million USD for other pharynx cancer to 508 million USD for breast cancer (Table 1). The top five cancers in terms of both number of research and funding amounts were breast cancer, tracheal, bronchus and lung cancer, liver cancer, colon and rectum cancer, and stomach cancer. The mean funding per project varied from 56,056 USD (SD 29,089 USD) for testicular cancer to 277,816 USD (SD 817,180 USD) for oesophageal cancer. The median ranged from 42,134 USD (IQR 38,140–57,361 USD) for mesothelioma to 82,263 USD (IQR 45,842–150,331 USD) for liver cancer.

**Table 1:** South Korean governmental research funds for 25 types of cancer in 2005–2017

Cancer	Number of research projects^a^ (% of total)	Sum of funding amount (USD) (% of total)	Mean (SD)	Median (IQR)	Max. of funding amount (USD)	Min. of funding amount (USD)
Bladder cancer	224 (1.3)	45,175,920 (1.6)	201,678 (413,219)	54,152 (43,054–172,356)	3,092,142	6279
Brain and nervous system cancer	208 (1.2)	41,801,454 (1.5)	200,969 (428,524)	75,958 (46,126–150,727)	3,092,142	4419
Breast cancer	3012 (17.4)	507,877,006 (18.3)	168,618 (388,459)	61,022 (43,445–155,674)	12,317,632	4308
Cervical cancer	476 (2.8)	67,038,896 (2.4)	140,838 (332,582)	73,006 (43,085–135,808)	4,842,876	5484
Colon and rectum cancer	2253 (13.0)	359,683,255 (13.0)	159,646 (281,421)	66,322 (43,318–156,691)	4,369,711	4305
Oesophageal cancer	89 (0.5)	24,725,583 (0.9)	277,816 (817,180)	51,891 (39,173–73,057)	3,670,566	4419
Gallbladder and biliary tract cancer	140 (0.8)	22,411,781 (0.8)	160,084 (216,101)	59,738 (42,893–130,102)	902,437	9024
Kidney cancer	153 (0.9)	16,967,505 (0.6)	110,899 (142,109)	53,058 (43,085–91,051)	723,826	6834
Larynx cancer	16 (0.1)	1,531,930 (0.1)	95,746 (127,238)	47,196 (24,748–97,779)	542,804	3905
Leukaemia	698 (4.0)	78,708,162 (2.8)	112,762 (184,828)	53,874 (41,995–107,246)	1,917,928	4419
Lip and oral cavity cancer	277 (1.6)	30,685,586 (1.1)	110,778 (173,473)	51,891 (43,085–88,958)	1,335,557	9024
Liver cancer	2121 (12.3)	359,910,785 (13.0)	169,689 (310,499)	82,263 (45,842–150,331)	3,092,142	3610
Malignant skin melanoma	326 (1.9)	53,382,377 (1.9)	163,750 (747,416)	47,655 (40,813–89,339)	13,058,884	8843
Mesothelioma	20 (0.1)	1,404,068 (0.1)	70,203 (64,989)	42,134 (38,140–57,361)	246,862	7533
Multiple myeloma	134 (0.8)	18,070,326 (0.7)	134,853 (360,193)	56,592 (43,796–106,566)	3,892,824	7032
Nasopharynx cancer	5 (0.03)	538,418 (0.02)	107,684 (82,799)	68,936 (36,278–165,965)	240,709	26,529
Non-Hodgkin lymphoma	287 (1.7)	37,178,554 (1.3)	129,542 (249,306)	63,487 (44,208–130,912)	3,388,511	6834
Other pharynx cancer	4 (0.02)	315,457 (0.01)	78,864 (69,995)	55,689 (18,967–115,586)	188,381	15,698
Ovarian cancer	619 (3.6)	75,159,865 (2.7)	121,421 (198,334)	53,812 (42,097–98,950)	2,044,770	5484
Pancreatic cancer	519 (3.0)	89,509,485 (3.2)	172,465 (237,389)	73,691 (43,946–192,220)	1,658,059	6834
Prostate cancer	886 (5.1)	138,598,295 (5.0)	156,431 (330,671)	54,416 (43,318–118,166)	4,203,554	1204
Stomach cancer	2022 (11.7)	299,360,928 (10.8)	148,052 (256,031)	58,929 (43,226–141,070)	2,928,440	4421
Testicular cancer	9 (0.1)	504,503 (0.02)	56,056 (29,089)	46,620 (40,740–68,339)	104,656	18,019
Thyroid cancer	246 (1.4)	26,851,359 (1.0)	109,152 (180,892)	53,002 (43,709–94,947)	1,541,824	4419
Tracheal, bronchus and lung cancer	2550 (14.7)	470,609,400 (17.0)	184,553 (385,722)	70,744 (44,292–160,571)	5,541,543	3889

^a^Multiple counting was allowed for the research projects that can be utilised in several types of cancers, accounting for 38.9% of the total number of research projects

The majority of the investment has been focused on basic research, accounting for 66.9% of total numbers and 47.9% of total funding (Table 2). As for mean funding per project, development research received the highest mean funding (295,433 USD (SD 385,352 USD)), followed by applied research (216,320 USD (SD 460,530 USD)) and basic research (114,523 USD (SD 260,770 USD)).

**Table 2:** South Korean governmental research funds by research stage in 2005–2017

	Number of research projects (% of total)	Sum of funding amount (USD) (% of total)	Mean (SD)	Median (IQR)	Max. of funding amount (USD)	Min. of funding amount (USD)
Basic research	11,573 (66.9)	1,325,369,180 (47.9)	114,523 (260,770)	51,494 (42,234–90,237)	12,317,632	2152
Applied research	3003 (17.4)	649,609,750 (23.5)	216,320 (460,530)	88,741 (49,883–197,342)	13,058,884	3889
Development research	2474 (14.3)	730,902,298 (26.4)	295,433 (414,082)	165,336 (75,243–333,566)	5,541,543	1204
Unable to specify	244 (1.4)	62,119,668 (2.2)	254,589 (385,352)	107,093 (n/a)	2,282,328	3610

Stomach cancer had the highest incidence in 2003 (44.7) while colon and rectum cancer had the highest incidence in 2013 (50.1) (Additional file 2: Table S2). Tracheal, bronchus and lung cancer had the highest mortality (30.1 (2003), 35.2 (2013)) in both years. Liver cancer had the highest YLLs (709.3) in 2003 while tracheal, bronchus and lung cancer had the highest YLLs (674.6) in 2013. The cancer type with the highest DALYs in 2003 was stomach cancer (721.1) and in 2013 it was tracheal, bronchus and lung cancer (686.8). In terms of burden changes from 1990 to 2006, the highest increase in DALYs was found in thyroid cancer (3.166) and the highest decrease in DALYs was found in testicular cancer (− 0.431) (Additional file 2: Table S2).

In the correlation analysis of four burden measures — incidence, mortality, YLLs and DALYs, all four burden measures were highly correlated with each other in each year of the analysis (correlation coefficients (R) of 0.87–0.99) (Additional file 3: Table S3). We chose to use DALYs as a representative burden measure in further analyses in this study to ensure consistency of comparison with many other previous studies which likewise applied DALYs as a burden indicator [22, 25, 28, 30].

The types of cancer that received the highest public interest as measured by web search intensity in the periods of analysis were gallbladder and biliary tract cancer, followed by liver cancer and cervical cancer. The lowest levels of public interest were shown for nasopharynx cancer, mesothelioma, and lip and oral cavity cancer (Additional file 4: Table S4).

In the univariate regression analysis using DALYs as an independent variable in each single period, the South Korean government’s funding from 2005 to 2007 was associated with DALYs in 2003 (R = 0.792, *P* <0.001), funding from 2008 to 2010 was associated with DALYs in 2006 (R = 0.610, *P* <0.001), funding from 2011 to 2013 was associated with DALYs in 2009 (R = 0.601, *P* = 0.001), and funding from 2015 to 2017 was associated with DALYs in 2013 (R = 0.823, *P* <0.001) (Table 3). Similarly, governmental funding was associated with public interest, as measured in web search intensity, in each corresponding year except for the funding period from 2008 to 2010 and the web search in 2006. Spearman’s rank correlation analysis also showed the research funding was associated with DALYs or web search intensities for all periods of analyses, respectively (Additional file 5: Table S5).

**Table 3:** Association of the South Korean governmental research funds with disability-adjusted life-years (DALYs) or web search intensity

Research funds by types of cancer	The measures of disease burden or public interest	Correlation coefficient (r)	*P* value
Sum of 2005–2007	DALYs (2003)	0.792	< 0.001
Sum of 2008–2010	DALYs (2006)	0.610	0.001
Sum of 2011–2013	DALYs (2009)	0.601	0.001
Sum of 2015–2017	DALYs (2013)	0.823	< 0.001
Change in funding amounts (from 2005 to 2007 to 2015–2017)	Change in DALYs (from 1990 to 2006)	0.184	0.378
Sum of 2005–2007	Web Search (2004)	0.717	< 0.001
Sum of 2008–2010	Web Search (2006)	0.261	0.207
Sum of 2011–2013	Web Search (2009)	0.748	< 0.001
Sum of 2015–2017	Web Search (2013)	0.548	0.005

The results of the sensitivity analysis, excluding multiply counted research projects which accounted for 38.9% of total cancer research in 2005–2017, show that DALYs and web search intensity is associated with research funding in each period of analysis, respectively, and the correlation coefficients of the relationship were somewhat lowered when compared to the results when we adopted multiple counting (Additional file 7: Table S7). This indicates that allowing multiple counting did not significantly affect the results of the analyses performed in our study.

We performed multivariable regression analysis using DALYs and web search intensity to investigate the influence of two variables on research funding (Additional file 6: Table S6). First, as a result of examining the multicollinearity, we confirmed that the variation inflation factor was less than 2.6 over all four periods, so that there was no multicollinearity between the two variables. As shown in Additional file 6: Table S6, DALYs had a significant effect on research funding over all four periods. On the other hand, web search had a significant effect only in two periods of analysis (2005–2007 and 2011–2013). Moreover, the increase in explanatory power (R^2^) resulting from adding public interest (web search intensity) as another variable was not noteworthy, i.e. the South Korean government’s research funding can be mostly explained by DALYs variable. Therefore, only DALYs was used for further analyses in this study.

**Fig. 1:**
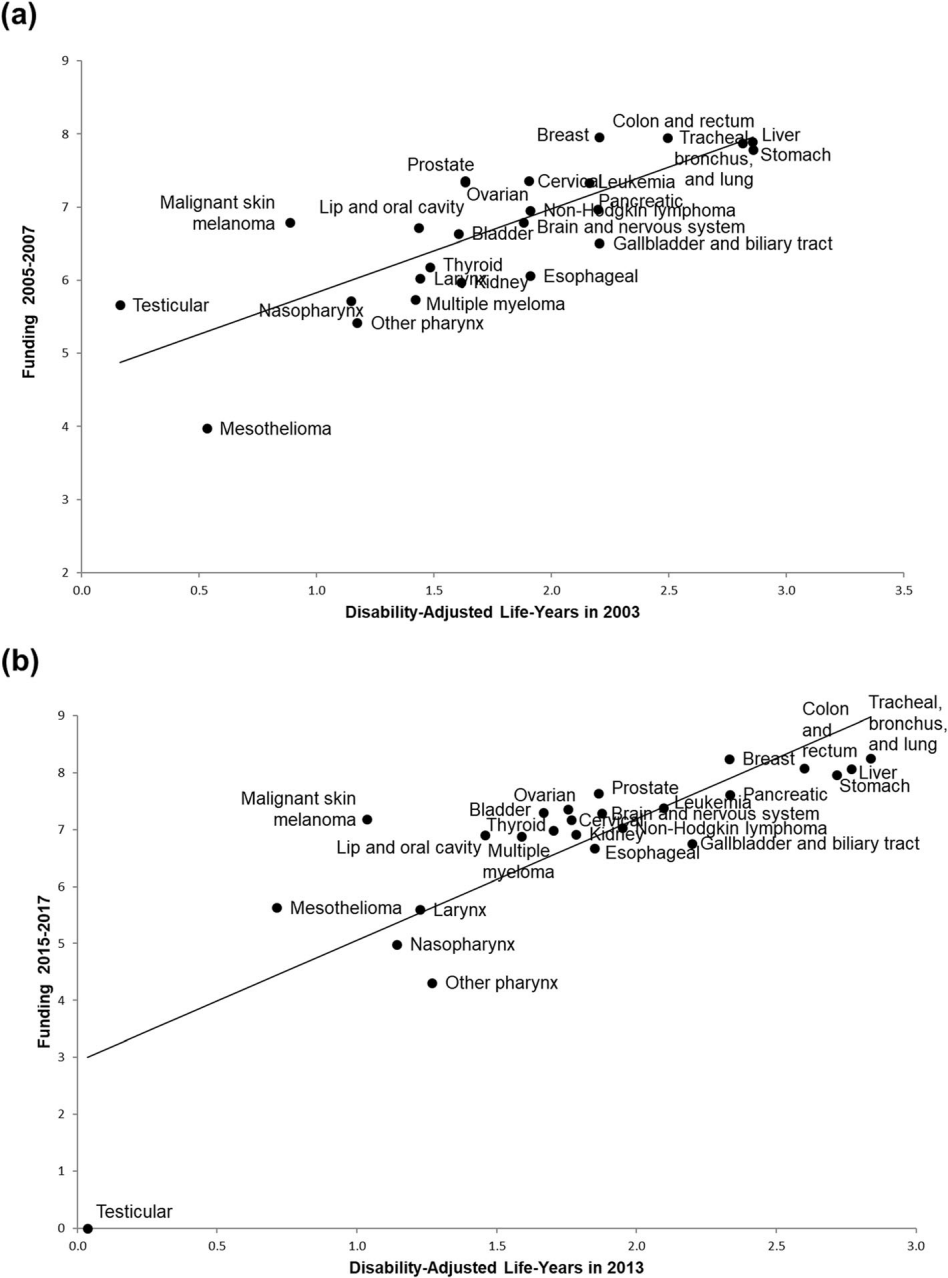
Relationship between government research funding and disability-adjusted life-years (DALYs) in Korea for 25 cancer types. **a** Funding during 2005–2007 and DALYs in 2003, **b** funding during 2015–2017 and DALYs in 2013. The line represents the predicted funding based on univariate linear regression with DALYs as the explanatory variable

**Fig. 2:**
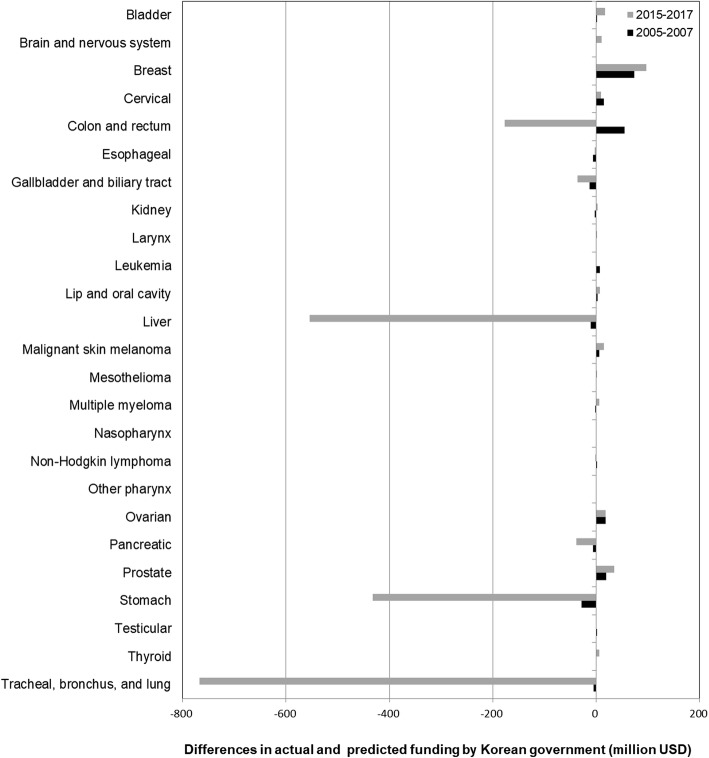
Differences of actual and expected funding for 25 cancer types applying DALYs as a predictor in 2005–2007 (black) and in 2015–2017 (grey)

The predicted funding as a function of DALYs was compared with the actual funding data (Fig. 1). The line represents the level of counterfactual funding that would be expected if DALYs were applied as the sole criterion for funding allocation. Based on this criterion, some cancers showed overfunding compared to the expected amount, while some cancers showed underfunding compared to the expected amount. Figure 2 shows the comparison of actual and counterfactual funding for each cancer when DALYs were applied as an explanatory variable. The results showed that stomach cancer received the least funding relative to the counterfactual funding amount in 2005–2007 while tracheal, bronchus and lung cancer received the least funding in 2015–2017. Breast cancer was the most funded cancer relative to the counterfactual funding in both the periods of 2005–2007 and 2015–2017.

In order to examine whether long-term changes in burden measures, not simply the burden values for single years, were adequately reflected in the funds allocation, we analysed the correlation between the burden change from 1990 to 2006 and the funding change over the next decade (from 2005–2007 to 2015–2017). The changes in the funding amounts were not associated with preceding changes in DALYs (R = 0.184, *P* = 0.378) (Table 3).

In addition, to examine whether the South Korean government’s funding allocations effectively reflected the discrepancies between the burden values specific to Korea and global values, we also analysed the correlation between Korea and global DALYs differences and South Korean research funding. However, the differences in DALYs were not associated with the funding level during the corresponding years, except for the funding period of 2005–2007 and DALYs in 2003 (Table 4).

**Table 4:** Differences in South Korean and global disability-adjusted life-years (DALYs) values and association with South Korean governmental research funds

Cancer		Differences in DALYs (Korean – Global)^a^			
		2003	2006	2009	2013
Bladder cancer		−3.38	−1.97	−0.43	1.84
Brain and nervous system cancer		− 35.57	− 37.55	− 36.54	−36.49
Breast cancer		−51.57	−37.37	−16.19	−4.63
Cervical cancer		−28.21	−34.72	−37.08	− 44.13
Colon and rectum cancer		87.63	123.68	156.63	162.55
Oesophageal cancer		−66.02	−60.20	−54.48	−52.30
Gallbladder and biliary tract cancer		118.01	117.77	119.50	114.97
Kidney cancer		2.62	7.36	12.92	19.20
Larynx cancer		−15.34	−19.58	−22.23	−24.66
Leukaemia		−40.02	−38.28	−31.21	−36.44
Lip and oral cavity cancer		−29.71	−32.06	−33.57	− 35.73
Liver cancer		452.03	425.15	397.77	331.61
Malignant skin melanoma		−13.03	−12.16	−10.96	− 10.39
Mesothelioma		−4.66	−4.09	−3.66	−3.50
Multiple myeloma		0.62	4.02	8.21	10.23
Nasopharynx cancer		−13.24	−11.70	− 11.43	−12.52
Non-Hodgkin lymphoma		−3.83	−2.64	−1.79	−0.81
Other pharynx cancer		−18.79	−18.44	− 19.56	−20.67
Ovarian cancer		−10.03	−6.79	−2.44	−0.16
Pancreatic cancer		64.18	74.62	91.20	105.41
Prostate cancer		−35.63	−28.57	−20.59	−12.46
Stomach cancer		421.41	380.21	346.85	271.43
Testicular cancer		−4.21	−4.23	−4.17	−4.01
Thyroid cancer		17.24	26.09	33.97	35.88
Tracheal, bronchus and lung cancer		165.47	179.38	189.14	180.83
Association with Korean governmental research funds	R	0.415	0.331	0.328	0.345
	*P* value	0.039	0.106	0.109	0.091

^a^The values of DALYs difference are shown as DALYs per 100,000 population

## Discussion

In this study, we analysed the relationship between government research funding and disease burden in Korea, particularly focusing on cancers. We also evaluated whether the research budget allocation properly reflected the changes in the burden and the burden levels specific to South Korea.

Various factors may influence the government’s healthcare research funding, including social, political, technological and economic (industrial) variables in addition to the disease burden variable. In previous studies, it was found that public interest and charity revenue could have significant effects on governmental budget allocation [25, 40]. In developed countries such as the United States and the United Kingdom, in particular, the charitable sector contributes largely to healthcare funding. However, in the case of South Korea, research support from the charitable sector accounts for only a small portion of total research investment in Korea [47]. The number of charitable organisations in Korea supporting academic research and scholarship amounted to about 2400 as of 2017, giving a total of 750 million USD [48]. Moreover, most of these organisations were devoted to supporting scholarship and it is difficult to calculate precisely how much of these grants have been invested particularly in cancer research. Although there are relevant research foundations, such as the Korea Cancer Research Foundation, the annual support amounts to several millions of dollars or less. Given this situation in Korea, we can conclude that the role of the public sector, such as the government-sponsored programmes, has been far more significant than that of the charitable sector.

In addition, industrial variables, such as the investments of pharmaceutical and/or medical companies, may affect national research funding. However, it is difficult to obtain accurate data on funding from private corporations by types of cancer, since this information is very rarely available in the public domain. It might be one of the reasons why preceding studies did not contain industrial factors in their analyses of the relationship. Unfortunately, this data is not available in South Korea either. Therefore, our analyses include two independent variables – disease burden and public interest – on which we have data that is precise, reliable and easily accessible.

With regards to DALYs as a predictor, a correlation coefficient (R) of 0.82 in 2015–2017 was higher than the values of previous studies. In the study by Gross et al. [22], an R of 0.62 was obtained for NIH funding (1996) and DALYs (1990), and in more recently reported findings by Gillum et al. [25], an R of 0.57 was obtained for NIH funding (2006) and DALYs (2004). In the case of Norway, an R of 0.62 for national research investments (2012) and DALYs (2010) was obtained [28], and in the case of China, an R of 0.40 was obtained for the National Natural Science Foundation of China funding (2012) and DALYs (2010) [30]. (In Xu et al.’s study of China [30], only the funding amounts were log-transformed for analysis. To perform our comparison under the same conditions, with all variables analysed in logarithmic scale, we converted the original DALYs values presented in their study to a logarithmic scale and then applied these values to regression analysis. The resulting R was 0.59, which was lower than our results.) These results demonstrate that South Korea’s allocation of research funds more appropriately reflected the disease burden compared to other countries in which the same analysis was performed. Since only a few studies have been implemented to date, similar analyses from many other countries should be encouraged so that we can track each country’s research investment trends. This will enable us to perform follow-up comparative analysis that will extend our understanding of the relationship between research funds and disease burden levels.

Although the allocation of research funds in South Korea reflected the absolute values of the cancer burden in a particular given year fairly well, the allocation did not reflect the changes in the burden. Specifically, budget allocations over the last decade did not properly reflect the long-term burden changes that preceded the allocations. Since one of the main goals of governmental research support is to prevent an increase in disease burden [49, 50], if the burden for a certain disease increases rapidly, it should be given a greater allocation in funding compared to a disease showing a decrease in burden. However, as shown in Table 3, the increase or decrease in disease burden levels were not reflected in the changes of the funding amounts.

Several cancers that had previously been overfunded, such as breast cancer, prostate cancer, ovarian cancer and cervical cancer, still received greater funds than the expected amount after 10 years, while some cancers that were previously underfunded, such as stomach cancer, gallbladder and biliary tract cancer, and liver cancer, remained underfunded compared to expected values. The reason why overfunded cancers were given much larger amounts of funding for the last decade may be that certain types of cancer associated with femininity/masculinity, due to their sites of occurrence in the human body, tend to receive more social interest and concern. As a result, the heightened public awareness would result in these types of cancer receiving greater funds. In the case of underfunded cancers such as lung cancer and liver cancer, patients tend to be blamed by others who cite the patient’s smoking or drinking behaviors. This ‘blame the victim’ attitude [51] might have been a factor that reduced public investment. This is consistent with previous studies that explained the reasons for the higher/lower levels of funding for certain type of cancers [35, 37].

Previous studies revealed that hematological cancers such as leukaemia have received higher level of funding compared to their burden values in many countries [30, 35, 36, 40]. Notably, in our analysis of South Korea, although leukaemia received more funding compared to burden values in the past (2005–2007), the margin of overfunding was not large. Moreover, leukaemia received funding levels that almost corresponded to its burden values in recent years (2015–2017) (4.2% less funding than the expected amounts by DALYs measure), indicating an appropriate level of investment in leukaemia in consideration of its burden values.

In terms of the change in the status of funding (overfunding/underfunding) compared with the predicted funding, colon and rectum cancer showed the most significant change, shifting from being overfunded in 2005–2007 (+54.8 million USD) to being underfunded in 2015–2017 (−177.6 million USD) relative to the expected funding amount. More specifically, the value of DALYs for colon and rectum cancer showed the greatest increase among the 25 cancer types we analysed, from 311.3 in 2003 to 398.6 in 2013, but the funding amounts did not much increase in relation to the increase in DALYs. On the contrary, brain and nervous system cancer showed the most significant change from being underfunded in 2005–2007 (−784,786 USD) to being overfunded in 2015–2017 (+10.8 million USD) compared to the expected values. Specifically, the funding for brain and nervous system cancer increased greatly in the last decade, from 6.1 million USD to 19.2 million USD, but DALYs remained at similar values (76.2 in 2003 and 75.2 in 2013).

Due to the unique characteristics of disease burden in individual countries, country-specific diseases need to receive more attention and support from the government, unlike private sector investments that target the global market. The types of cancer with the highest differences in DALYs when we compared South Korean values with global values were liver cancer, stomach cancer, and tracheal, bronchus and lung cancer. However, the government’s investments have not appropriately reflected these discrepancies in the last 10 years, as shown in Table 4. Policy-makers and research funders should take these conditions into consideration when prioritising research projects, in keeping with the government’s responsibility to improve national health. There may be differing views on whether a country’s research should focus more on national or global disease burdens. An argument can also be made that the global disease profile also needs to be considered in public research investment, since the results of healthcare research can have global effects and benefit people suffering from various diseases around the world.

The Korean government has selected and fostered the healthcare sector as one of the major research investment areas, and various national plans and strategies (such as The 3rd Basic Biotechnology Promotion Plan (‘17-‘26), The 2nd National Infectious Diseases Technology Development Strategy (‘17-‘21), and Bio/Health Industry Innovation Strategy (‘19)) have been established and implemented [52]. These recent plans are aimed at improving public health and the quality of people’s lives to achieve a healthier and more vibrant life for the population. However, research investments in Korea in the past were more focused on the areas where economic and technological achievements (e.g. market growth and expansion, international trade balance improvement, strengthening technology competitiveness, patents and paper publication, etc.) can be rapidly produced [53] and very little consideration is given to the burden of diseases. In addition, the process of prioritising research projects had relied considerably on the subjective and qualitative judgements of expert committees [54] and recently began to introduce quantitative and objective data-based decision-making processes. However, as described above, research funding of the Korean government showed a higher correlation with cancer burden than other countries, the information on disease burden (specially burden change, country-specific diseases, and long-term overfunding or underfunding) needs to be considered more carefully in the healthcare research prioritisation process.

Our study has several limitations. First, even though it was able to include all national research projects conducted in Korea by using NTIS as the source of our data, the funding data may have contained research projects that are not obviously related to cancer, since the research projects listed in NTIS are not classified by disease types or categories. We identified research projects on cancer primarily by conducting keyword searches on research information using search fields (research title, research objective, research keyword and research summary), assuming that research on a particular type of cancer would cite the terms for that cancer in these fields. However, our data may have included irrelevant research projects if the terms related to a particular cancer were mentioned in the fields for tenuous reasons, to exaggerate the project’s association with cancer or to emphasise the importance of the research project. In this regard, after obtaining NTIS data, we further screened this using other fields such as ‘classification of science and technology’ and ‘classification of application’, in order to improve the accuracy of the data. For instance, if the classification of science and technology was ‘construction’ or ‘defence’ or ‘marketing,’ the project was excluded. Likewise, if the classification of application was ‘transportation’ or ‘art’ or ‘energy,’ the project was also excluded from our funding dataset. Although we performed additional screening and carefully reviewed the data qualitatively, our data may have still contained noise. Secondly, our analyses examined only types of cancers, not all diseases or conditions. In order to derive all-round meaningful implications for public health improvement in Korea and compare our findings with other countries’ health research investments, there will need to be additional comprehensive analyses that encompass more types of diseases. Thirdly, other burden indicators, such as prevalence, hospital admissions, medical care costs, etc., were not covered in this study. Since analysis results can vary depending on which burden indicators are applied, a wider range of disease burden measures will need to be analysed collectively to obtain more reliable results. In addition, various other variables that are likely to affect research funding, such as quality of life considerations, exciting/promising developments in the field, local expertise in an area, advocacy groups etc., were not included in our analysis due to the difficulties of obtaining reliable data. Further studies that identify meaningful influence factors will help enrich our knowledge and implications for healthcare research funding.

## Conclusions

To the best of our knowledge, this is the first study identifying the relationship between government research funding and cancer burdens (DALYs) in South Korea. In South Korea, the funding allocations corresponded more closely to DALYs than in other countries. However, this study found that the South Korean government has not properly reflected long-term burden changes and has not invested more research funds to the types of cancer that have higher burden values in Korea compared to the global values. Although perfect alignment between funds and disease burdens could be controversial and many factors should be considered simultaneously in the process of national budget allocation, quantitative and statistical analyses that reflect public health conditions need to be considered in the decision-making process as one of the fundamental criteria for determining research priorities.

## Supplementary information

**Additional file 1: Table S1.** The keywords for searching research projects by 25 types of cancer.

**Additional file 2: Table S2.** The values of disease burden in sigular year (2003 and 2013) and the value changes in DALYs from 1990 to 2006 in South Korea for 25 types of cancer.

**Additional file 3: Table S3.** The correlation analysis of four disease burden measures (incidence, mortality, YLLs and DALYs) in each year of the analysis. (a) 2003, (b) 2006, (c) 2009 and (d) 2013.

**Additional file 4: Table S4.** The web search intensities by 25 types of cancer in each year of the analysis.

**Additional file 5: Table S5.** Spearman’s rank correlation of the South Korean governmental research funds with DALYs or web search intensity.

**Additional file 6: Table S6.** Multivariable regression of the South Korean governmental research funds as predicted by DALYs and web search intensity.

**Additional file 7: Table S7.** Sensitivity analysis excluding multiply counted research projects.

## Data Availability

The datasets generated and analysed in this study are available from the authors on reasonbale request.

## Abbreviations

DALYs: Disability-adjusted life years
GBD: Global Burden of Disease
GDP: Gross domestic product
KRW: Korean Won
NIH: National Institutes of Health
NTIS: National Science & Technology Information Service
R&D: Research and development
SD: Standard deviation
USD: United States Dollars
YLLs: Years of life lost
