# Supplementary material for: The relationship between government research funding and the cancer burden in South Korea: implications for prioritising health research
Source: Health Res Policy Syst. 2019 Dec 23;17:103. doi: 10.1186/s12961-019-0510-6 (PMC6929284; doi:10.1186/s12961-019-0510-6)
Supplement: Supplementary file 1 — Additional file 1: Table S1. The keywords for searching research projects by 25 types of cancer. [file 12961_2019_510_MOESM1_ESM.docx]

**Additional file for**

**The relationship between government research funding and the cancer burden in South Korea: Implications for prioritizing health research**

**Table S1. The keywords for searching research projects by 25 types of cancer.**

| Cancer | Search keywords |
| --- | --- |
| Bladder cancer | “bladder cancer” |
| Brain and nervous system cancer | “brain cancer”, “nervous system cancer” |
| Breast cancer | “breast cancer” |
| Cervical cancer | “cervical cancer” |
| Colon and rectum cancer | “colon cancer”, “rectum cancer” |
| Esophageal cancer | “esophageal cancer” |
| Gallbladder and biliary tract cancer | “gallbladder cancer”, “biliary tract cancer”, “bile duct cancer”, “cholangiocarcinoma” |
| Kidney cancer | “kidney cancer”, “renal cell carcinoma” |
| Larynx cancer | “larynx cancer” |
| Leukemia | “leukemia” |
| Lip and oral cavity cancer | “lip cancer”, “oral cavity cancer”, “oral cancer”, “tongue cancer”, “periodontal cancer”, “gingiva carcinoma” |
| Liver cancer | “liver cancer” |
| Malignant skin melanoma | “malignant skin melanoma”, “malignant melanoma” |
| Mesothelioma | “mesothelioma” |
| Multiple myeloma | “multiple myeloma” |
| Nasopharynx cancer | “nasopharynx cancer” |
| Non-Hodgkin lymphoma | “non-Hodgkin lymphoma” |
| Other pharynx cancer | “pharynx cancer” |
| Ovarian cancer | “ovarian cancer” |
| Pancreatic cancer | “pancreatic cancer” |
| Prostate cancer | “prostate cancer” |
| Stomach cancer | “stomach cancer”, “gastric cancer” |
| Testicular cancer | “testicular cancer”, “testis cancer” |
| Thyroid cancer | “thyroid cancer” |
| Tracheal, bronchus, and lung cancer | “tracheal cancer”, “bronchus cancer”, “bronchogenic carcinoma”, “lung cancer” |
