# Supplementary material for: The relationship between government research funding and the cancer burden in South Korea: implications for prioritising health research
Source: Health Res Policy Syst. 2019 Dec 23;17:103. doi: 10.1186/s12961-019-0510-6 (PMC6929284; doi:10.1186/s12961-019-0510-6)
Supplement: Supplementary file 2 — Additional file 2: Table S2. The values of disease burden in sigular year (2003 and 2013) and the value changes in DALYs from 1990 to 2006 in South Korea for 25 types of cancer. [file 12961_2019_510_MOESM2_ESM.docx]

**Additional file for**

**The relationship between government research funding and the cancer burden in South Korea: Implications for prioritizing health research**

**Table S2. The values of disease burden in sigular year (2003 and 2013) and the value changes in DALYs from 1990 to 2006 in South Korea for 25 types of cancer.**

| Cancer | Measures of Disease Burden in South Korea | | | | | | | | |
| --- | --- | --- | --- | --- | --- | --- | --- | --- | --- |
|  | Rate^a^(rank) | | | | | | | | |
|  | Incidence | | Mortality | | YLLs | | DALYs | | Change in DALYs |
|  | 2003 | 2013 | 2003 | 2013 | 2003 | 2013 | 2003 | 2013 | 1990-2006 |
| Bladder cancer | 6.1(11) | 8.2(11) | 2.1(13) | 2.7(13) | 37.0(16) | 41.8(16) | 40.3(16) | 46.3(17) | 0.482(12) |
| Brain and nervous system cancer | 4.6(14) | 7.6(12) | 2.1(14) | 2.3(14) | 74.1(12) | 71.2(10) | 76.2(12) | 75.2(10) | -0.101(19) |
| Breast cancer | 20.0(5) | 34.5(5) | 4.9(7) | 7.1(7) | 144.8(7) | 190.1(6) | 159.2(6) | 214.4(6) | 0.469(13) |
| Cervical cancer | 8.4(8) | 6.7(13) | 2.9(10) | 2.2(15) | 75.8(11) | 54.9(14) | 80.1(11) | 58.4(14) | -0.163(21) |
| Colon and rectum cancer | 29.8(3) | 50.1(1) | 13.0(4) | 18.3(4) | 296.2(4) | 371.8(4) | 311.3(4) | 398.6(4) | 0.972(5) |
| Esophageal cancer | 4.2(16) | 4.8(16) | 3.6(9) | 3.3(11) | 80.0(9) | 69.2(11) | 81.2(10) | 70.8(12) | -0.151(20) |
| Gallbladder and biliary tract cancer | 9.0(7) | 11.9(8) | 7.6(5) | 8.6(6) | 157.9(5) | 155.6(7) | 159.8(5) | 158.3(7) | 0.441(15) |
| Kidney cancer | 4.3(15) | 6.7(14) | 1.6(15) | 2.7(12) | 39.7(14) | 58.3(13) | 41.3(15) | 60.7(13) | 1.540(2) |
| Larynx cancer | 3.5(17) | 3.0(19) | 1.2(17) | 0.8(21) | 25.6(18) | 14.9(21) | 27.6(18) | 16.8(21) | -0.352(24) |
| Leukemia | 5.1(12) | 6.2(15) | 3.8(8) | 4.2(8) | 142.2(8) | 122.0(8) | 144.6(8) | 125.0(8) | -0.319(23) |
| Lip and oral cavity cancer | 2.6(19) | 3.3(18) | 1.1(19) | 1.2(19) | 26.0(17) | 27.2(19) | 27.1(19) | 28.7(19) | 0.414(16) |
| Liver cancer | 28.0(4) | 35.7(4) | 25.7(3) | 23.7(2) | 709.3(1) | 576.2(2) | 715.8(2) | 585.4(2) | 0.289(18) |
| Malignant skin melanoma | 1.2(21) | 2.4(21) | 0.3(23) | 0.4(23) | 7.1(23) | 9.7(23) | 7.7(23) | 10.8(23) | 0.582(10) |
| Mesothelioma | 0.2(25) | 0.3(25) | 0.1(24) | 0.2(24) | 3.4(24) | 5.0(24) | 3.4(24) | 5.2(24) | 0.457(14) |
| Multiple myeloma | 1.5(20) | 2.8(20) | 1.1(18) | 1.8(17) | 25.5(19) | 36.9(18) | 26.3(20) | 38.7(18) | 0.942(6) |
| Nasopharynx cancer | 0.7(23) | 0.6(23) | 0.5(22) | 0.5(22) | 13.7(22) | 13.6(22) | 14.0(22) | 13.9(22) | 0.782(7) |
| Non-Hodgkin lymphoma | 5.0(13) | 8.4(10) | 2.8(11) | 3.6(10) | 79.3(10) | 85.3(9) | 81.3(9) | 88.8(9) | 0.396(17) |
| Other pharynx cancer | 1.1(22) | 1.7(22) | 0.6(21) | 0.8(20) | 14.5(21) | 18.0(20) | 14.9(21) | 18.6(20) | 1.199(3) |
| Ovarian cancer | 2.8(18) | 3.9(17) | 1.5(16) | 2.1(16) | 41.1(13) | 54.3(15) | 42.9(13) | 56.8(15) | 0.712(9) |
| Pancreatic cancer | 7.2(9) | 11.3(9) | 7.0(6) | 10.4(5) | 156.3(6) | 213.5(5) | 157.8(7) | 215.8(5) | 0.533(11) |
| Prostate cancer | 6.7(10) | 16.7(7) | 2.3(12) | 4.1(9) | 38.4(15) | 61.9(12) | 42.8(14) | 73.0(11) | 1.130(4) |
| Stomach cancer | 44.7(1) | 47.4(2) | 29.3(2) | 23.1(3) | 708.4(2) | 506.0(3) | 721.1(1) | 521.3(3) | -0.296(22) |
| Testicular cancer | 0.4(24) | 0.5(24) | 0.0(25) | 0.0(25) | 1.2(25) | 0.8(25) | 1.5(25) | 1.1(25) | -0.431(25) |
| Thyroid cancer | 11.7(6) | 24.6(6) | 1.0(20) | 1.6(18) | 24.4(20) | 37.8(17) | 30.4(17) | 50.4(16) | 3.166(1) |
| Tracheal, bronchus, and lung cancer | 32.8(2) | 46.2(3) | 30.1(1) | 35.2(1) | 642.9(3) | 674.6(1) | 650.5(3) | 686.8(1) | 0.712(9) |

^a^The definition of a rate for each disease burden is: (1) Incidence: Cases per 100,000 population, (2) Mortality: Deaths per 100,000 population, (3) YLLs: YLLs per 100,000 population, (4) DALYs: DALYs per 100,000 population (from http://ghdx.healthdata.org/gbd-results-tool).
