# Supplementary material for: The relationship between government research funding and the cancer burden in South Korea: implications for prioritising health research
Source: Health Res Policy Syst. 2019 Dec 23;17:103. doi: 10.1186/s12961-019-0510-6 (PMC6929284; doi:10.1186/s12961-019-0510-6)
Supplement: Supplementary file 3 — Additional file 3: Table S3. The correlation analysis of four disease burden measures (incidence, mortality, YLLs and DALYs) in each year of the analysis. (a) 2003, (b) 2006, (c) 2009 and (d) 2013. [file 12961_2019_510_MOESM3_ESM.docx]

**Additional file for**

**The relationship between government research funding and the cancer burden in South Korea: Implications for prioritizing health research**

**Table S3. The correlation analysis of four disease burden measures (incidence, mortality, YLLs, and DALYs) in each year of the analysis. (a) 2003, (b) 2006, (c) 2009, and (d) 2013.**

| (a) 2003 |  |  |  |  |
| --- | --- | --- | --- | --- |
|  | Incidence | Mortality | YLLs | DALYs |
| Incidence | 1 |  |  |  |
| Mortality | 0.9194 | 1 |  |  |
| YLLs | 0.9129 | 0.9908 | 1 |  |
| DALYs | 0.9188 | 0.9908 | 0.9999 | 1 |
| (b) 2006 |  |  |  |  |
|  | Incidence | Mortality | YLLs | DALYs |
| Incidence | 1 |  |  |  |
| Mortality | 0.8943 | 1 |  |  |
| YLLs | 0.8908 | 0.9905 | 1 |  |
| DALYs | 0.8996 | 0.9903 | 0.9998 | 1 |
| (c) 2009 |  |  |  |  |
|  | Incidence | Mortality | YLLs | DALYs |
| Incidence | 1 |  |  |  |
| Mortality | 0.8703 | 1 |  |  |
| YLLs | 0.8712 | 0.9904 | 1 |  |
| DALYs | 0.8833 | 0.9900 | 0.9996 | 1 |
| (d) 2013 |  |  |  |  |
|  | Incidence | Mortality | YLLs | DALYs |
| Incidence | 1 |  |  |  |
| Mortality | 0.8655 | 1 |  |  |
| YLLs | 0.8712 | 0.9910 | 1 |  |
| DALYs | 0.8852 | 0.9903 | 0.9995 | 1 |
