# Supplementary material for: The relationship between government research funding and the cancer burden in South Korea: implications for prioritising health research
Source: Health Res Policy Syst. 2019 Dec 23;17:103. doi: 10.1186/s12961-019-0510-6 (PMC6929284; doi:10.1186/s12961-019-0510-6)
Supplement: Supplementary file 4 — Additional file 4: Table S4. The web search intensities by 25 types of cancer in each year of the analysis. [file 12961_2019_510_MOESM4_ESM.docx]

**Additional file for**

**The relationship between government research funding and the cancer burden in South Korea: Implications for prioritizing health research**

**Table S4. The web search intensities by 25 types of cancer in each year of the analysis.**

| Cancer | 2004^*^ | 2006 | 2009 | 2013 |
| --- | --- | --- | --- | --- |
| Bladder cancer | 455 | 360 | 158 | 143 |
| Brain and nervous system cancer | 183 | 46 | 39 | 57 |
| Breast cancer | 446 | 402 | 290 | 529 |
| Cervical cancer | 537 | 440 | 291 | 600 |
| Colon and rectum cancer | 333 | 220 | 200 | 344 |
| Esophageal cancer | 408 | 280 | 166 | 155 |
| Gallbladder and biliary tract cancer | 491 | 647 | 595 | 418 |
| Kidney cancer | 245 | 106 | 98 | 132 |
| Larynx cancer | 123 | 142 | 118 | 140 |
| Leukemia | 556 | 431 | 236 | 250 |
| Lip and oral cavity cancer | 109 | 33 | 44 | 138 |
| Liver cancer | 674 | 490 | 300 | 464 |
| Malignant skin melanoma | 100 | 104 | 124 | 72 |
| Mesothelioma | 0 | 0 | 208 | 26 |
| Multiple myeloma | 335 | 134 | 185 | 62 |
| Nasopharynx cancer | 100 | 50 | 0 | 43 |
| Non-Hodgkin lymphoma | 391 | 520 | 413 | 289 |
| Other pharynx cancer | 0 | 358 | 0 | 68 |
| Ovarian cancer | 292 | 243 | 157 | 176 |
| Pancreatic cancer | 457 | 370 | 238 | 439 |
| Prostate cancer | 190 | 249 | 194 | 268 |
| Stomach cancer | 480 | 388 | 317 | 458 |
| Testicular cancer | 191 | 146 | 136 | 77 |
| Thyroid cancer | 133 | 86 | 82 | 183 |
| Tracheal, bronchus, and lung cancer | 474 | 338 | 314 | 396 |

^*^The web search intensity in 2004 was applied instead of that of 2003 since Google Trends are available from 2004.
