# Supplementary material for: The relationship between government research funding and the cancer burden in South Korea: implications for prioritising health research
Source: Health Res Policy Syst. 2019 Dec 23;17:103. doi: 10.1186/s12961-019-0510-6 (PMC6929284; doi:10.1186/s12961-019-0510-6)
Supplement: Supplementary file 5 — Additional file 5: Table S5. Spearman’s rank correlation of the South Korean governmental research funds with DALYs or web search intensity. [file 12961_2019_510_MOESM5_ESM.docx]

**Additional file for**

**The relationship between government research funding and the cancer burden in South Korea: Implications for prioritizing health research**

**Table S5. Spearman’s rank correlation of the South Korean governmental research funds with DALYs or web search intensity.**

| Research funds by types of cancer | The measures of disease burden or public interest | Spearman’s rho (*p*-value) |
| --- | --- | --- |
| Sum of 2005-2007 | DALYs (2003) | 0.792 (<0.001) |
| Sum of 2008-2010 | DALYs (2006) | 0.848 (<0.001) |
| Sum of 2011-2013 | DALYs (2009) | 0.749 (<0.001) |
| Sum of 2015-2017 | DALYs (2013) | 0.794 (<0.001) |
| Sum of 2005-2007 | Web Search (2004) | 0.605 (0.001) |
| Sum of 2008-2010 | Web Search (2006) | 0.558 (0.004) |
| Sum of 2011-2013 | Web Search (2009) | 0.541 (0.005) |
| Sum of 2015-2017 | Web Search (2013) | 0.676 (<0.001) |
