# Supplementary material for: The relationship between government research funding and the cancer burden in South Korea: implications for prioritising health research
Source: Health Res Policy Syst. 2019 Dec 23;17:103. doi: 10.1186/s12961-019-0510-6 (PMC6929284; doi:10.1186/s12961-019-0510-6)
Supplement: Supplementary file 6 — Additional file 6: Table S6. Multivariable regression of the South Korean governmental research funds as predicted by DALYs and web search intensity. [file 12961_2019_510_MOESM6_ESM.docx]

**Additional file for**

**The relationship between government research funding and the cancer burden in South Korea: Implications for prioritizing health research**

**Table S6. Multivariable regression of the South Korean governmental research funds as predicted by DALYs and web search intensity.**

| Research funds by types of cancer | The measures of disease burden or public interest | β | *p-*value | adjR^2^ | F  (*p*-value) |
| --- | --- | --- | --- | --- | --- |
| Sum of 2005-2007 | DALYs (2003) | 0.565 | 0.001 | 0.693 | 28.120 (<0.001) |
|  | Web Search (2004) | 0.379 | 0.014 |  |  |
| Sum of 2008-2010 | DALYs (2006) | 0.687 | 0.003 | 0.328 | 6.862 (0.005) |
|  | Web Search (2006) | -0.134 | 0.52 |  |  |
| Sum of 2011-2013 | DALYs (2009) | 0.354 | 0.016 | 0.634 | 21.777 (<0.001) |
|  | Web Search (2009) | 0.603 | <0.001 |  |  |
| Sum of 2015-2017 | DALYs (2013) | 1.002 | <0.001 | 0.67 | 25.418 (<0.001) |
|  | Web Search (2013) | -0.231 | 0.228 |  |  |
