# Supplementary material for: The relationship between government research funding and the cancer burden in South Korea: implications for prioritising health research
Source: Health Res Policy Syst. 2019 Dec 23;17:103. doi: 10.1186/s12961-019-0510-6 (PMC6929284; doi:10.1186/s12961-019-0510-6)
Supplement: Supplementary file 7 — Additional file 7: Table S7. Sensitivity analysis excluding multiply counted research projects. [file 12961_2019_510_MOESM7_ESM.docx]

**Additional file for**

**The relationship between government research funding and the cancer burden in South Korea: Implications for prioritizing health research**

**Table S7. Sensitivity analysis excluding multiply counted research projects.**

| Research funds by types of cancer | The measures of disease burden or public interest | Correlation Coefficient (r) | *p*-value |
| --- | --- | --- | --- |
| Sum of 2005-2007 | DALYs (2003) | 0.548 | 0.005 |
| Sum of 2008-2010 | DALYs (2006) | 0.751 | <0.001 |
| Sum of 2011-2013 | DALYs (2009) | 0.671 | <0.001 |
| Sum of 2015-2017 | DALYs (2013) | 0.735 | <0.001 |
| Change in funding amounts (from 2005-2007 to 2015-2017) | Change in DALYs  (from 1990 to 2006) | 0.016 | 0.940 |
| Sum of 2005-2007 | Web Search (2004) | 0.680 | <0.001 |
| Sum of 2008-2010 | Web Search (2006) | 0.511 | 0.009 |
| Sum of 2011-2013 | Web Search (2009) | 0.671 | <0.001 |
| Sum of 2015-2017 | Web Search (2013) | 0.546 | 0.005 |
